# A probabilistic approach to enhance the efficiency of case finding in hospital quality management: A case study using readmissions

**DOI:** 10.1371/journal.pone.0341187

**Published:** 2026-01-27

**Authors:** Michael M. Havranek, Aleksandra Bosancic, Esther Ammann, Balthasar L. Hug

**Affiliations:** 1 Competence Center for Health Data Science, Faculty of Health Sciences and Medicine, University of Lucerne, Lucerne, Switzerland; 2 Department of Internal Medicine, Cantonal Hospital Lucerne, Lucerne, Switzerland; 3 Faculty of Health Sciences and Medicine, University of Lucerne, Lucerne, Switzerland; Yonsei University Medical Center: Yonsei University Health System, KOREA, REPUBLIC OF

## Abstract

Hospital readmissions prolong patient suffering and increase healthcare expenditures. Unplanned readmission rates, such as those reported by the Center for Medicare & Medicaid Services (CMS), distinguish between planned and unplanned readmissions. However, within unplanned readmissions, there is no distinction between those that are preventable versus unpreventable by the hospitals. Alternative approaches attempting to identify potentially preventable readmissions directly from coded medical data have been explored but have shown low sensitivity. Consequently, identifying preventable readmissions remains a time-consuming task for hospital quality managers seeking to allocate improvement resources effectively. To address this challenge, we aimed to develop and evaluate a probabilistic approach to improve the identification of preventable readmissions among unplanned readmissions. Using a retrospective record review of 600 single inpatient stays from a tertiary referral hospital group in Switzerland, we investigated the hypothesis that readmitted patients with a low expected probability for readmission (based on a logistic regression model using patient characteristics) would retrospectively show higher odds of having experienced a potentially or most likely preventable readmission (as assessed by the reviewers). The results confirmed our hypothesis: patients in the third with the lowest expected probability of readmission (compared with those in the highest third) had 6.6 and 8.7 times higher odds, respectively, of having experienced a potentially or most likely preventable readmission. Among preventable readmissions, the leading causes of readmission were surgical complications, medication-related reasons, nonsurgical complications, and premature discharge. Our proposed probabilistic approach can be used by hospital quality managers to focus case finding efforts on unexpectedly readmitted patients and aid effective resource allocation for improvement initiatives.

## Introduction

Hospital readmissions prolong patient suffering and increase healthcare expenditures, which is why readmission rates are used in quality monitoring and pay-for-performance initiatives in many countries (see, e.g., [1]). In a well-known example, unplanned readmission rates that distinguish between planned and unplanned readmissions within 30 days are reported by the American Center for Medicare & Medicaid Services (CMS) [1,2]. These unplanned readmissions focus on readmissions that arose from acute clinical events requiring urgent rehospitalization and were not planned or foreseen during the index hospitalization. However, they do not indicate which of these are preventable by the hospitals (i.e., could have been avoided if different treatment decisions had been made), even though this distinction is crucial to plan and implement targeted improvement efforts. Other approaches that attempt to classify readmissions as potentially preventable based on coded medical data have been explored [3–5] but have shown low sensitivity [6].

International research suggests that about one fifth of all hospital patients are readmitted within 30 days of discharge [7]. Recent reports from Switzerland show that depending on the exact patient population between 2.8 and 14.4% of unplanned readmissions occur [8]. Of these, between 11 and 13% may be preventable, given the results of previous research [9,10]. Thus, case finding of preventable readmissions within unplanned readmissions is a time-consuming task for hospital quality managers who are trying to allocate improvement resources effectively [11].

To address this challenge, our goal was to develop and evaluate a probabilistic approach to retrospectively determine unplanned readmissions with increased odds of having been preventable. Our approach divides readmitted patients based on their expected probability of experiencing an unplanned readmission, using only patient characteristics from the risk-adjustment models of the readmission rates. We hypothesized that readmitted patients with a *low* expected probability for readmission would show *higher* odds of their readmissions having been preventable (e.g., because of unexpected complications of care). Although this hypothesis may be counterintuitive at first glance, it stems from the simple stochastic fact that readmitted patients with a low expected probability for readmission (based on their patient characteristic) must, on average, have a higher likelihood of having experienced an unexpected adverse event or care coordination issue compared to readmitted patients with an already preexisting high expected readmission risk. To our best knowledge, this hypothesis has never been scientifically investigated, so we tested it across four patient samples in a retrospective record review study.

## Materials and methods

### Study design and data

We conducted a quantitative retrospective record review study at a tertiary referral hospital group with multiple sites in Switzerland. The hospital provided administrative medical data to identify unplanned readmissions using a version of the CMS algorithm adapted for the Swiss medical coding system (see more detailed information below).

The administrative dataset [12] contained all inpatient stays treated by the hospital group during the five-year study period of 2018–2022, with up to 50 diagnosis codes for each stay (from the International Statistical Classification of Diseases and Related Health Problems, 10th revision, German Modification, ICD-10-GM [13]), up to 100 procedure codes (from the Swiss classification of surgical interventions, CHOP [13]), the diagnosis-related group (from the SwissDRG system [14]), other clinically relevant variables such as admission and discharge conditions, and patients’ demographic information. Unplanned readmissions were flagged according to the CMS definitions (version 2020 [15–18]), which were translated and modified for the Swiss medical coding system, as proposed in [19], described in [8], and validated in [6]. See Part A of the S1 Appendix for a brief comparison between the original CMS method and our adapted version.

Electronic medical records were retrospectively accessed directly by the reviewers from the hospital group during the study period from July 4, 2022 until March 30, 2024 and contained all available information from the patient documentation (e.g., discharge letters, reports, notes, charts, medications, imaging, laboratory, and other results). The study was approved through a jurisdictional inquiry by the Ethics Committee Northwest & Central Switzerland (May 19, 2022; ID: Req-2022–00615). Informed consent was not required, as the study was considered a quality control project and because the researchers that analyzed the data received only anonymized data that did not contain information to identify individual patients.

### Sampling and record review

A random sample of pairs of inpatient stays was drawn (each comprising an index hospitalization and a readmission flagged as either planned or unplanned) from eligible hospitalizations based on the CMS inclusion/exclusion criteria [15–18]. Two typical medical and two surgical patient populations were selected from among the patient populations that CMS covers to include a diverse set of patients: readmissions following acute myocardial infarction (AMI), pneumonia (PN), or elective total hip and knee arthroplasty (THATKA), and readmissions in a general surgical/gynecologic cohort of the hospital-wide sample (SURG).

In total, 600 single stays, or 300 pairs comprising an index hospitalization and readmission within the same hospital, were reviewed by four reviewers, consisting of medical doctors and a medical student in the last year of medical school. All reviewers underwent standardized training to familiarize themselves with the definitions of unplanned and preventable readmissions and to learn the structured review process. Their assessments were collected using a standardized online questionnaire that was developed and applied previously [6]. See Part B of the S1 Appendix for a short translation of the main contents of the online questionnaire. As the retrospective reviewer judgment may introduces subjectivity, 30 of the 300 pairs were reviewed by two independent reviewers to assess the inter-rater reliability (IRR). Disagreements were resolved in discussions.

For each case pair, the reviewers assessed whether the readmission was unplanned, potentially preventable, or most likely preventable. For potentially or most likely preventable readmissions, the reviewers assessed the cause of readmission according to a previously developed classification framework (initially developed and used in [4] and subsequently employed in adapted form in [6]). In addition, the reviewers were asked to provide their level of subjective certainty about each question they answered (e.g., “How certain are you about this decision?”) based on a Likert scale ranging from 1 (“very uncertain”) to 10 (“very certain”).

A readmission was defined as “unplanned” if it arose from acute clinical events requiring urgent rehospitalization (i.e., was not planned or foreseen during the index hospitalization [15]) and as “potentially preventable” if it was related to any condition treated during the index hospitalization (see, e.g., [4]). “Most likely preventable” readmissions were those judged by the reviewers as preventable if different treatment decisions had been made (e.g., where the performed treatment had not followed clinical guidelines).

### Statistical analysis

The goal of the statistical analysis was to test whether our hypothesis is correct that readmitted patients with a low expected probability for readmission would show higher odds of their readmissions having been preventable. As the first stage of the statistical analyses, the frequency and cause of readmissions judged by the reviewers as unplanned, potentially preventable, and most likely preventable were assessed across the four included patient populations (AMI, PN, THATKA, and SURG). The entire sample was then divided into thirds based on the expected probability of experiencing an unplanned readmission, as estimated by the risk-adjustment model. We used the patient variables from the risk-adjustment model proposed by CMS that is directed toward the hospital-wide population and uses only patient characteristics (but no information on treatment decisions or complications of care [16,20]). As previously adapted to the Swiss setting and annually employed in national quality monitoring since (see, e.g., [8]), it was recalculated across the eligible hospitalizations of the participating hospital group and achieved an area under the receiver operating curve (AUC) value of 0.7 on the data from the participating hospital, indicating moderate predictive performance.

Next, Fisher’s exact tests with odds ratios (ORs) and 95% confidence intervals (CIs) of having experienced either a potentially preventable or most likely preventable readmission were calculated between the third of patients with the lowest probability for an unplanned readmission and the third of patients with the highest probability. In the case of most likely preventable readmissions, a pseudo-count of 1 was added to the entire contingency table to avoid infinite values due to the division by zero in the case of zero counts (see, e.g., [21] for a discussion). Finally, a sensitivity analysis using the median and quartiles (instead of tertiles) to divide the readmitted patients was conducted to verify the robustness of the results.

Notably, reviewers were blinded regarding patients’ expected probability of unplanned readmission. All statistical analyses were performed in Python (version 3.8.8) and results were considered statistically significant if p < 0.05, after adjusting the p value threshold for multiple comparisons across the two outcomes of potentially vs. most likely preventable readmissions using the Hochberg method (which is a sharper Bonferroni procedure [22]).

## Results

### Frequency and distribution

7,512 (5.7%) out of 132,472 hospitalizations during 2018–2022 were flagged as unplanned readmissions according to our version of the unplanned readmissions algorithm (see Methods section). Resource restrictions allowed us to examine 300 case pairs (i.e., 600 individual cases) of index hospitalization and readmission (271 flagged as unplanned and 29 as planned). They were randomly selected for review across 4,019 readmissions (7.5%) from the four investigated patient populations (i.e., 75 per population from a total of 196 AMI readmissions, 222 PN readmissions, 106 THATKA readmissions, and 3,495 SURG readmissions; see Table 1). The positive predictive value (PPV = true positives/ (true positives + false positives)) of the algorithm for correctly flagging unplanned readmissions was 94.1% across the entire sample.

**Table 1:** Unplanned vs. planned readmissions as flagged by the unplanned readmissions method and as assessed by the reviewers.

	Cases	Flagged		Reviewed	
		Unplanned	Planned	Unplanned	Planned
**AMI**	75	63	12	58	17
**PN**	75	75	0	74	1
**THATKA**	75	70	5	72	3
**SURG**	75	63	12	63	12
**TOTAL**	300	271	29	267	33

*Note:* The distribution of readmissions flagged as unplanned vs. planned was neither held constant nor stratified across the four patient populations and does not, therefore, reflect the actual proportions of unplanned vs. planned readmissions in the underlying populations. “Flagged” = as flagged by the unplanned readmissions method, “Reviewed” = as assessed by the reviewers, AMI = acute myocardial infarction patients, PN = pneumonia patients, THATKA = total hip or total knee arthroplasty patients, SURG = general surgical/gynecologic cohort patients, and TOTAL = all four patient populations combined.

Reviewers reported high certainty in distinguishing between planned and unplanned readmissions (mean Likert score = 9.9, SD = 0.5), unpreventable and potentially preventable readmissions (mean = 8.4, SD = 1.4), and potentially preventable and most likely preventable readmissions (mean = 8.5, SD = 1.3). The IRR between the reviewers was 96.7% for planned vs. unplanned, 92.9% for unpreventable vs. potentially preventable, and 82.1% for potentially vs. most likely preventable readmissions.

Table 2 shows the distribution of cases assessed by the reviewers as potentially preventable and most likely preventable. Of the 267 unplanned readmissions (as assessed by the reviewers), 156 (58.1%) were judged as potentially preventable, and among those, 10 (6.5%) were reckoned as most likely preventable. Potentially preventable readmissions were more common in surgical populations (THATKA and SURG), whereas most likely preventable readmissions were comparably distributed across the patient populations.

**Table 2:** Frequency of potentially and most likely preventable readmissions within the unplanned readmissions (as assessed by the reviewers).

	Cases	Unplanned	Unpreventable	Pot. Prev.	N (%)	M.-L. Prev.	N (%)
**AMI**	75	58	26	32	(55.2%)	2	(6.3%)
**PN**	75	74	45	29	(39.2%)	3	(10.3%)
**THATKA**	75	72	19	53	(73.6%)	2	(3.8%)
**SURG**	75	63	22	41	(65.1%)	3	(7.3%)
**TOTAL**	300	267	112	155	(58.1%)	10	(6.5%)

*Note:* Readmissions that were assessed as most likely preventable are a subset of the readmissions that were assessed as potentially preventable. “Pot. Prev.” = number of potentially preventable readmissions (expressed as a percentage in parentheses), “N (%)” = number (percentage) of cases, “M.-L. Prev.” = number of most likely preventable readmissions (expressed as a percentage in parentheses), AMI = acute myocardial infarction patients, PN = pneumonia patients, THATKA = total hip or total knee arthroplasty patients, SURG = general surgical/gynecologic cohort patients, and TOTAL = all four patient populations combined.

### Causes of readmission

Table 3 illustrates the causes of readmission (as assessed by the reviewers) for cases judged as at least potentially preventable. The leading causes of preventable readmissions were surgical complications (47.7%), medication-related reasons (14.2%), nonsurgical complications (11.0%), and premature discharge (9.0%). The causes of most likely preventable readmissions were readmissions not justified according to medical criteria (40.0%), those with missing or erroneous diagnosis or therapy during the index hospitalization (40.0%), and premature or other inadequate discharge during the initial stay (10.0% and 10.0%, respectively). Among these, readmissions not justified from a medical standpoint were always considered as most likely preventable (100.0%) and readmissions with missing or erroneous diagnosis or therapy in 80.0% (see Table 3).

**Table 3:** Frequency and preventability of the causes of preventable readmissions (as assessed by the reviewers).

	Pot. Prev.	N (%)	M.-L. Prev.	N (%)	Preventability in %
**Surgical complications**	74	(47.7%)	0	(0.0%)	0.0%
**Medication-related reasons**	22	(14.2%)	0	(0.0%)	0.0%
**Nonsurgical complications**	17	(11.0%)	0	(0.0%)	0.0%
**Premature discharge**	14	(9.0%)	1	(10.0%)	7.1%
**Relapse or aggravation of known affection**	8	(5.2%)	0	(0.0%)	0.0%
**Missing or erroneous diagnosis or therapy**	5	(3.2%)	4	(40.0%)	80.0%
**Other reasons**	5	(3.2%)	0	(0.0%)	0.0%
**Readmission not justified from medical standpoint**	4	(2.6%)	4	(40.0%)	100.0%
**Inadequate patient behavior**	3	(1.9%)	0	(0.0%)	0.0%
**Other inadequate discharge**	2	(1.3%)	1	(10.0%)	50.0%
**Failure of post-discharge follow-up care**	1	(0.6%)	0	(0.0%)	0.0%
**Full sample**	155	(100.0%)	10	(100.0%)	6.5%

*Note:* “Pot. Prev.” = number of potentially preventable readmissions with the indicated causes (expressed as a percentage in parentheses), “N (%)” = number (percentage) of cases, “M.-L. Prev.” = number of most likely preventable readmissions with the indicated causes (expressed as a percentage in parentheses), “Preventability in %” = number of most likely preventable readmissions expressed as a percentage of the number of potentially preventable readmissions with the indicated causes, and “Full sample” = all causes combined.

### Testing the main hypothesis

Regarding our main hypothesis, the average expected probability of unplanned readmission was 3.2% in the third of readmitted patients with the lowest probability for readmission, and 18.7% in the third of patients with the highest probability. The OR of having experienced a potentially preventable readmission was 6.6 (CI = 3.4–12.8, p < 0.001) for the third of readmitted patients with the lowest vs. highest expected probability for unplanned readmission. Consistently, the third with the lowest expected probability of unplanned readmission was more likely to have experienced a most likely preventable readmission than the highest third (OR = 8.7, CI = 1.1–70.8, p = 0.035). These results confirm our hypothesis. In addition, our sensitivity analysis showed that the results were not dependent on the exact cutoff thresholds, as comparable results were obtained when using the median (for potentially preventable: OR = 3.4, CI = 2.0–5.6, p < 0.001; for most likely preventable: OR = 5.3, CI = 1.1–24.6, p = 0.035) or quartiles (for potentially preventable: OR = 12.5, CI = 5.5–28.5, p < 0.001; for most likely preventable: OR = 5.3, CI = 0.6–46.7, p = 0.208) to divide the readmitted patients instead of tertiles.

## Discussion

This study evaluated a probabilistic approach to improve the efficiency of retrospective case finding, using the identification of preventable readmissions among patients with unplanned readmissions as an example. We found that readmitted patients in the third with the lowest expected probability of experiencing an unplanned readmission had 6.6 times higher odds of having experienced a potentially preventable and 8.7 times higher odds of having experienced a most likely preventable readmission compared to the third with the highest expected probability. As discussed below, these findings may be used by hospital quality managers to allocate improvement resources more effectively.

### Interpretation of the main findings

Among unplanned readmissions, 58.1% were judged as potentially preventable by the reviewers. Among these, 6.5% were judged as most likely preventable. These findings are in line with a previous study [9] where 47% of all-cause readmissions were considered as potentially preventable, of which 11% were judged as very or completely preventable. Comparable results were documented in another report [10] that judged 13% of unplanned readmissions as preventable. In addition, potentially preventable readmissions were more frequent in the surgical populations (THATKA and SURG), which was consistent with previous observations [23] that general surgical readmissions were more frequently considered as preventable than general medical and geriatric readmissions.

In terms of the reasons for potentially preventable readmission, we found that surgical complications were the most frequent cause, followed by medication-related reasons, nonsurgical complications, and premature discharge. Similar findings were made by colleagues [5] who showed that the most common causes of unforeseen readmissions (beside relapse or aggravation of a previously known condition) were complications of surgical care, followed by drug-related adverse events, failure of post-discharge follow-up, and inadequate or premature discharge.

The reasons for readmission judged most frequently by the reviewers as most likely preventable were readmissions that were not justified according to medical criteria, readmissions with missing or erroneous diagnosis or therapy, and premature or other inadequate discharge during the index hospitalization. Comparable observations were made previously [10], showing that the most reported causes of preventable readmissions were diagnostic (e.g., misdiagnosis/ delayed diagnosis), management (e.g., inadequate discharge or transition of care issues), and medication issues (e.g., incorrect prescription or use).

### Practical relevance, limitations, and conclusion

From a practical perspective, our main results of higher odds of preventable readmissions in unexpectedly readmitted patients are important because unplanned readmission rates are widely used to compare quality of care across hospitals (see, e.g., [1,2]). For reliable statistical comparisons, sufficient caseloads are necessary, which requires the investigation of large patient populations (such as AMI, PN, etc.). However, if hospitals have lower than average results in these statistical comparisons, they are confronted with many unplanned readmissions and do not know how to prioritize case finding or where to allocate improvement resources.

Risk-adjustment models are designed to correct hospital quality comparisons for the fact that certain patients are more likely to experience readmissions. However, as shown here, the expected probabilities from these models can also be used to make case finding more efficient by letting hospital quality managers focus on cases where a readmission was not expected. In practice, the herein suggested approach could be implanted as follows: First, hospital quality managers would focus on quality indicators where comparisons with other healthcare providers showed above average results for their hospitals using large sample sizes that allow for valid and reliable statistical comparisons. Second, from among all the observed quality-related events in this quality indicator (in our case, all unplanned readmissions), they would preselect the third (or fourth, or fifth) of events with the lowest expected probability of the event in question. Finally, these selected cases can be analyzed by the treating clinicians in a retrospective record review to identify reoccurring preventable reasons for the occurrence of the quality-related events.

We confirmed our hypothesis that potentially and most likely preventable readmissions are more frequently found in patients who were unexpectedly readmitted. With that, we provide hospital quality managers with a strategy to make case finding more efficient. More specifically, if hospital quality managers focus their case finding on readmitted patients with a low expected probability for readmission, their odds of finding potentially preventable or even most likely preventable readmissions are significantly higher.

Our findings are in line with an earlier study [24] showing that analyzing low-risk patient populations allows for better discrimination between high- and low-performing hospitals. Thus, we believe that our approach may also be beneficial to enhance the case finding in other quality indicators (like mortality or complication rates). In addition, our approach may complement alternative strategies that attempt to focus case findings on particular patient populations, such as early readmissions (within, e.g., seven days instead of 30 days [25–27]).

This study has the following key limitation: Our results were assessed using unplanned readmission rates based on the methodology proposed by CMS, focused on four specific patient populations from a limited sample of stays, and were evaluated in a single-site analysis only. Therefore, it is possible that our findings cannot be generalized to different quality indicators, patient populations, and other hospitals or countries. Future research should, therefore, replicate our findings using other quality indicators, larger and more diverse patient populations, and healthcare settings and possibly investigate complementary strategies to improve the efficiency of case finding that may be combined with our probabilistic approach.

In conclusion, we have developed and evaluated a probabilistic approach to make case finding more efficient for hospital quality managers. By focusing retrospective record reviews on readmitted patients from the third with the lowest expected probability of readmission, quality managers have 6.6 and 8.7 times higher odds, respectively, of finding readmissions that were potentially or most likely preventable, compared with the third of readmitted patients with the highest expected probability. The implication of our findings is that the efficiency of case finding preventable adverse events (in our case readmissions) retrospectively can be optimized by focusing on patients with a low preexisting risk for a given adverse event. There is more future research warranted but our findings may be used by hospital quality managers or policymakers to allocate quality improvement initiatives more effectively.

## Supporting information

S1 Appendix Part A: Comparison of the original CMS method of identifying unplanned readmissions with our Swiss-adapted version. Part B: Translation of the main content of the online questionnaire (DOCX)
